# Integrated likelihood for phylogenomics under a no-common-mechanism model

**DOI:** 10.1186/s12864-020-6608-y

**Published:** 2020-04-16

**Authors:** Hunter Tidwell, Luay Nakhleh

**Affiliations:** Department of Computer Science, Rice University, Houston, TX USA

**Keywords:** Phylogenomics, Multispecies coalescent, No common mechanism, Integrated likelihood

## Abstract

**Background:**

Multi-locus species phylogeny inference is based on models of sequence evolution on gene trees as well as models of gene tree evolution within the branches of species phylogenies. Almost all statistical methods for this inference task assume a common mechanism across all loci as captured by a single value of each branch length of the species phylogeny.

**Results:**

In this paper, we pursue a “no common mechanism" (NCM) model, where every gene tree evolves according to its own parameters of the species phylogeny. Based on this model, we derive an analytically integrated likelihood of both species trees and networks given the gene trees of multiple loci under an NCM model. We demonstrate the performance of inference under this integrated likelihood on both simulated and biological data.

**Conclusions:**

The model presented here will afford opportunities for exploring connections among various criteria for estimating species phylogenies from multiple, independent loci. Furthermore, further development of this model could potentially result in more efficient methods for searching the space of species phylogenies by focusing solely on the topology of the phylogeny.

## Background

**Fig. 1:**
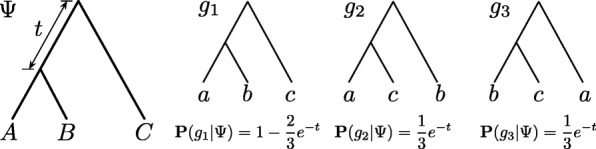
The multispecies coalescent (MSC) model. The species tree *Ψ* defines a probability distribution on gene tree topologies, as shown for the three gene trees on three taxa, where *t* is the branch length in coalescent units

A phylogenetic tree models the evolutionary history of a set of taxa (genes, species, etc.) from their most recent common ancestor. Analyses of genome-wide data from several groups of species have highlighted a significant phenomenon, namely the incongruence among phylogenetic trees of the different genomic regions as well as with the phylogeny of the species [1]. One cause of incongruence is incomplete lineage sorting, or ILS, which can be mathematically well understood under the *multispecies coalescent* (MSC) model [2]. Figure 1 illustrates the gene tree probability distribution that the multispecies coalescent defines.

However, as Maddison noted [1], other processes could give rise to incongruence among gene trees, including hybridization, which gives rise to phylogenetic networks [3]. The multispecies coalescent has been extended to incorporate such processes [4–7]. These findings have given rise to phylogenomics—the inference of a species phylogeny from genome-wide data. Given *m* gene trees ${\mathcal {G}} = \{g_{1},\ldots,g_{m}\}$ for *m* independent loci (genomic regions), the likelihood of a species phylogeny *Ψ* and its branch lengths *Λ* and inheritance probabilities *Γ* (more on these in the “Methods” section) is given by

1 $$  {\mathcal{L}}(\Psi, \Lambda, \Gamma | {\mathcal{G}}) = \prod_{i=1}^{m} \mathbf{P}(g_{i} | \Psi, \Lambda, \Gamma).  $$

Assuming, for example, that ILS is the sole cause of all incongruence among gene trees in ${\mathcal {G}}$, then **P**(*g*_*i*_|*Ψ*,**λ**) is given by MSC, as illustrated in Fig. 1. If both ILS and reticulation are at play, then the probability distribution is given by the multispecies network coalescent [4].

The formulation given by Eq. (1) assumes a “common mechanism" across all loci—all gene trees “grow" within a species tree given by a single setting of branch lengths (and, in the case of a phylogenetic network, a single setting of inheritance probabilities). In this paper we explore a “no common mechanism," or NCM, model, which states that each gene tree evolved within the branches of the species phylogeny under a totally separate process from all other gene trees as given by the parameters (e.g., branch lengths) of the species phylogeny. The main motivation behind this work is taming the complexity of statistical inference of phylogenetic networks. As calculating the likelihood of a phylogenetic network is computationally very expensive [8, 9], integrating out the continuous parameters and focusing the search on only the topologies could significantly reduce the computational requirements of phylogenetic network inference.

It is important to note that the NCM model has been explored and studied in the “classical" phylogeny problem (inferring a phylogenetic tree from a sequence alignment). Under that setting, NCM posits that each site in the sequence alignment evolved under its own branch lengths of the phylogenetic tree. Tuffley and Steel [10] established a seminal result in the field by proving that the maximum parsimony and maximum likelihood estimates of a phylogenetic tree are equal under an NCM model based on a symmetric Poisson process of nucleotide substitution. Additional mathematical results based on the NCM model were later established by Steel and Penny [11]. The NCM model allowed for analytically integrating out the branch lengths of a phylogenetic tree and efficiently exploring the space of phylogenetic trees [12]. Steel [13] showed that it is possible to achieve statistical consistency of inference under certain NCM models.

However, while the NCM model was used by some as an argument in favor of using maximum parsimony, Holder et al. [14] showed problems with this argument. Furthermore, Huelsenbeck et al. [15] argued that “biologically inspired phylogenetic models" outperform the NCM model. More specifically, it is hard to justify a phylogenetic model by which every site, including adjacent ones, has its own evolutionary process. This is why the NCM model was deemed more useful for morphological characters than molecular characters in a sequence alignment. An NCM model is more appropriate in phylogenomics, where different genomic regions could have evolved under different rates and even under different trees. This has led to the development of methods that partition the genomic data into regions, where the sites within each region are assumed to have evolved identically; e.g., see [16]. In this paper, we analytically derive an integrated likelihood model under the multispecies coalescent with the NCM, and show the performance of inference based on this NCM model on data simulated under common and no common mechanisms, as well as a biological data set. Like the argument made by Huelsenbeck et al. [12], the work we present here could lead to more efficient ways of exploring the space of species phylogenies and establishing connections between inference based on different models, including the parsimony formulation given by the “minimizing deep coalescences" criterion [1, 17, 18]).

## Methods

In order to account for both reticulation and incomplete lineage sorting, we use the phylogenetic network model since it generalizes trees. A *phylogenetic network* *Ψ* on set $\mathcal {X}$ of taxa is a rooted, directed, acyclic graph (DAG) with set of nodes *V*(*Ψ*)={*r*}∪*V*_*L*_∪*V*_*T*_∪*R*, where

- *indeg*(*r*)=0 (*r* is the *root* of *Ψ*);
- ∀*v*∈*V*_*L*_, *indeg*(*v*)=1 and *outdeg*(*v*)=0 (*V*_*L*_ are the *external tree nodes*, or *leaves*, of *Ψ*);
- ∀*v*∈*V*_*T*_, *indeg*(*v*)=1 and *outdeg*(*v*)≥2 (*V*_*T*_ are the *internal tree nodes* of *Ψ*); and,
- ∀*v*∈*R*, *indeg*(*v*)=2 and *outdeg*(*v*)=1 (*R* are the *reticulation nodes* of *Ψ*).

The set of edges *E*(*Ψ*)⊆*V*×*V* consists of *reticulation edges*, whose heads are reticulation nodes, and *tree edges*, whose heads are tree nodes. The leaves of the network are bijectively labeled by elements of $\mathcal {X}$.

We assume that we have *ℓ* reticulation nodes *R*={*r*_1_,…,*r*_*ℓ*_} with *ℓ* associated inheritance probabilities *γ*_1_,…,*γ*_*ℓ*_, respectively (that is, node *r*_*i*_ has two parents *pr*_*i*_ and $\overline {pr}_{i}$ with inheritance probabilities *γ*_*i*_ and (1−*γ*_*i*_) associated with the edges *b*1_*i*_=(*pr*_*i*_,*r*_*i*_) and $b2_{i}=(\overline {pr}_{i},r_{i})$, respectively). In addition to the topology of a phylogenetic network *Ψ*, each edge *b*=(*u*,*v*) in *E*(*Ψ*) has a length *λ*_*b*_ measured in coalescent units, which is the number of generations divided by effective population size on that branch. We use *Ψ* to refer to the topology of the phylogenetic network, *Λ* to refer to its branch lengths, and *Γ* to refer to the inheritance probabilities associated with all reticulation nodes. A species tree is a phylogenetic network with no reticulation nodes (and an empty *Γ*).

### Distribution of gene tree topologies

Given a phylogenetic network *Ψ*, its branch lengths *Λ* and inheritance probabilities *Γ* on the reticulation edges, the gene tree topology is a random variable whose probability mass function (pmf) we now briefly review. This pmf was originally derived for the case of species trees by Degnan and Salter [19] and later extended to the case of phylogenetic networks by Yu et al. [4].

We denote by *Ψ*_*u*_ the set of nodes that are reachable from the root of *Ψ* via at least one path that goes through node *u*∈*V*(*Ψ*). Then given a phylogenetic network *Ψ* and a gene tree *g* for some locus *j*, a coalescent history is a function *h*:*V*(*g*)→*E*(*Ψ*) such that the following two conditions hold:

- if *v* is a leaf in *g*, then *h*(*v*)=(*x*,*y*) where *y* is the leaf in *Ψ* with the label of the species from which the allele labeling leaf *v* in *G* is sampled; and,
- if *v* is a node in the subtree of *g* that is rooted at *u*, and *h*(*u*)=(*p*,*q*), then *h*(*v*)=(*x*,*y*) where *y*∈*Ψ*_*q*_.

Given a phylogenetic network *Ψ* and a gene tree *g* for locus *j*, we denote by *H*_*Ψ*_(*g*) the set of all coalescent histories of *g* within the branches of *Ψ*. Then the pmf of the gene tree is given by

2 $$  \mathbf{P}(g|\Psi,\Lambda,\Gamma)=\sum_{h \in H_{\Psi}(g)}\mathbf{P}(h|\Psi,\Lambda,\Gamma),  $$

where *Λ* are the branch lengths of the phylogenetic network (in coalescent units), *Γ* is the inheritance probabilities matrix, and **P**(*h*|*Ψ*,*Λ*,*Γ*) gives the pmf of the coalescent history random variable, which can be computed as

3 $$ {{{}\begin{aligned} \mathbf{P}(h|\Psi,\Lambda,\Gamma) = \frac{w(h)}{d(h)} \prod_{b \in E(\Psi)} \frac{w_{b}(h)}{d_{b}(h)} p_{u_{b}(h)v_{b}(h)}(\lambda_{b}) \prod_{r_{i} \in R} \gamma_{i}^{u_{b1_{i}}(h)} (1-\gamma_{i})^{u_{b2_{i}}(h)}. \end{aligned}}}  $$

In this equation, *u*_*b*_(*h*) and *v*_*b*_(*h*) denote the number of lineages that respectively enter and exit edge *b* of *Ψ* under coalescent history *h*. The term $p_{u_{b}(h)v_{b}(h)}(\lambda _{b})$ is the probability of *u*_*b*_(*h*) gene lineages coalescing into *v*_*b*_(*h*) during time *λ*_*b*_. And *w*_*b*_(*h*)/*d*_*b*_(*h*) is the proportion of all coalescent scenarios resulting from *u*_*b*_(*h*)−*v*_*b*_(*h*) coalescent events that agree with the topology of the gene tree. This quantity without the *b* subscript corresponds to the root of *Ψ*. Notice that removing the rightmost product over the reticulation nodes in Eq. (3) gives the pmf for species trees.

### An integrated likelihood framework

**Integrating out the branch lengths.** The function *p*_*uv*_(*t*) employed by Eq. (3) is given by

4 $$ {{{}\begin{aligned} p_{uv}(t) = \sum_{j=u}^{v} \left(e^{-\frac{j(j-1)}{2}t} \frac{(2j-1)(-1)^{j-v}}{v!(j-v)!(v+j-1)} \prod_{y=0}^{j} \frac{(v+y)(u-y)}{u+y}\right), \end{aligned}}}  $$

which, for simplifying the equations below, can be written as $p_{uv}(t) = \sum _{j=u}^{v} \left (e^{-\frac {j(j-1)}{2}t} \cdot f(u,v,j)\right)$, where $f(u,v,j) = \frac {(2j-1)(-1)^{j-v}}{v!(j-v)!(v+j-1)} \prod _{y=0}^{j} \frac {(v+y)(u-y)}{u+y}$. Assuming a truncated Exponential prior with support in (0,*τ*] and hyperparameter value of 1 on *t*, we have

5 $$  {}\begin{array}{lll} p_{uv}^{\tau} & = & \int_{0}^{\tau} p_{uv}(t) p(t) dt \\ & = & \int_{0}^{\tau} \left(\sum_{j=u}^{v} \left(e^{-\frac{j(j-1)}{2}t} \cdot f(u,v,j)\right) \right) \left(\frac{e^{-t}}{1-e^{-\tau}} \right) dt\\ & = & \frac{1}{1-e^{-\tau}} \sum_{j=u}^{v} f(u,v,j) \int_{0}^{\tau} e^{-(\frac{j(j-1)}{2}+1)t}dt \\ & = & \frac{1}{1-e^{-\tau}} \sum_{j=u}^{v} \left(f(u,v,j) \cdot \frac{2}{j(j-1)+2} \!\left(1\!-e^{-(\frac{j(j-1)}{2}+1)\tau}\right)\!\right). \\ \end{array}  $$

Using this result, and assuming all branch lengths of the species phylogeny are independent, we have

6 $$ {{{}\begin{aligned} \mathbf{P}(h|\Psi,\Gamma) &= \int_{0}^{\tau}\mathbf{P}(h|\Psi,\Lambda,\Gamma) p(\Lambda) d\Lambda\\ &= \frac{w(h)}{d(h)} \prod_{b \in E(\Psi)} \frac{w_{b}(h)}{d_{b}(h)} p^{\tau}_{u_{b}(h)v_{b}(h)} \prod_{r_{i} \in R} \!\gamma_{i}^{u_{b1_{i}}(h)} (1\,-\,\gamma_{i})^{u_{b2_{i}}(h)}. \end{aligned}}}  $$

**Integrating out the inheritance probabilities.** We now have $\mathbf {P}(h|\Psi) = \int \mathbf {P}(h|\Psi,\Gamma) p(\Gamma) d\Gamma $, where *p*(*Γ*) is a prior on the inheritance probabilities, and the multiple integration is taken over all *ℓ* gamma’s on [0,1]. We assume *γ*_*i*_∼*Beta*(2,2), so that we have a conjugate prior (pdf in this case is $\frac {\gamma _{i}(1-\gamma _{i})}{B(2,2)}$). Then, we have

7 $$ {}\begin{array}{lll} \mathbf{P}(h|\Psi) & = \frac{w(h)}{d(h)} \prod_{b \in E(\Psi)} \frac{w_{b}(h)}{d_{b}(h)} p^{\tau}_{u_{b}(h)v_{b}(h)}\\ &\hspace{29pt} \prod_{r_{i} \in R} \frac{1}{B(2,2)} \int_{0}^{1} \gamma_{i}^{u_{b1_{i}}(h)+1} (1\,-\,\gamma_{i})^{u_{b2_{i}}(h)+1}d\gamma_{i}\\ & = \frac{w(h)}{d(h)} \left(\prod_{b \in E(\Psi)} \frac{w_{b}(h)}{d_{b}(h)} p^{\tau}_{u_{b}(h)v_{b}(h)} \right) \left(\frac{1}{B(2,2)}\right)^{\ell}\\ & \hspace{29pt} \prod_{r_{i} \in R} \frac{(u_{b1_{i}}(h)+1)! (u_{b2_{i}}(h)+1)!}{(u_{b1_{i}}(h)+u_{b2_{i}}(h)+3)!}. \\ \end{array}  $$

Finally,

8 $$  \mathbf{P}(g|\Psi)=\sum_{h \in H_{\Psi}(g)}\mathbf{P}(h|\Psi),  $$

where *Ψ* is given by its topology alone.

Observe that if one treats the branch lengths and inheritance probabilities in Eq. (1) as a nuisance parameter, then the integrated likelihood is given by $\mathbf {P}({\mathcal {G}}|\Psi) = \int \int \left [ \prod _{i=1}^{m} \mathbf {P}(g_{i} | \Psi,\Lambda,\Gamma) p(\Lambda) p(\Gamma) d\Gamma d\Lambda \right ],$ where ${\mathcal {G}}=\{g_{1},g_{2},\ldots,g_{m}\}$, and **P**(*g*_*i*_|*Ψ*,**λ**) is computed as in [4, 20].

**Inference** The calculation given by Eq. (8) above allows us to compute

9 $$ {{{}\begin{aligned} f(\Psi | {\mathcal{G}}) = \prod_{i=1}^{m} \left[ \int_{\mathbf{\Lambda}} \int_{\mathbf{\Gamma}}\mathbf{P}(g_{i} | \Psi,\Lambda,\Gamma) p(\Lambda) p(\Gamma) d\Gamma d\Lambda \right] =\prod_{i=1}^{m} \mathbf{P}(g_{i} | \Psi) \end{aligned}}}  $$

from a set ${\mathcal {G}}$ of input gene trees inferred on multiple independent loci. For inferring an optimal network under Eq. (9), also known as the maximum integrated likelihood network, a search for the network *Ψ* that maximizes $f(\Psi | {\mathcal {G}})$ is conducted. Since no branch lengths or inheritance probabilities are optimized or sampled, just like the case of inference under the MDC criterion, we use the exact search heuristic and moves of [18].

**Efficiency** Observe that the computational requirements of calculating the probability according to Eq. (8) with the analytical integration of branch lengths and inheritance probabilities remain the same as those of computing the probability of a gene tree given a species network and its specific branch lengths and inheritance probabilities as in [4]. The major gain in computational requirements is in the search procedure. Search based on the integrated likelihood evaluates only topologies, and need not consider optimizing or sampling the continuous parameters. For example, for the case of a rooted species tree on three taxa, search based on the integrated likelihood only inspects three topologies; that is, likelihood calculations are done exactly three times to identify the optimal species tree. Searching for the maximum likelihood species tree while sampling branch lengths requires walk in the infinite space of branch length settings. It is important to note that the likelihood of different parameterizations of a given species tree (or network) is not a “nice" convex function. Therefore, searching for branch lengths and, in the case of phylogenetic networks, inheritance probabilities that maximize the likelihood is not a simple computational task and requires dealing with local maxima. Using integrated likelihood, it is even possible to evaluate phylogenetic networks with small numbers of taxa even exhaustively, a task that cannot be done once the branch lengths and inheritance probabilities are involved. For example, there are 105 rooted species trees on five taxa. Finding the one that maximizes the integrated likelihood based on the computations above can be done by exhaustively calculating the likelihoods for all 105 tree topologies. When branch lengths are included, searching the space while optimizing or sampling the branch lengths cannot be avoided.

## Results

### Accuracy on data simulated under a common mechanism

**Fig. 2:**
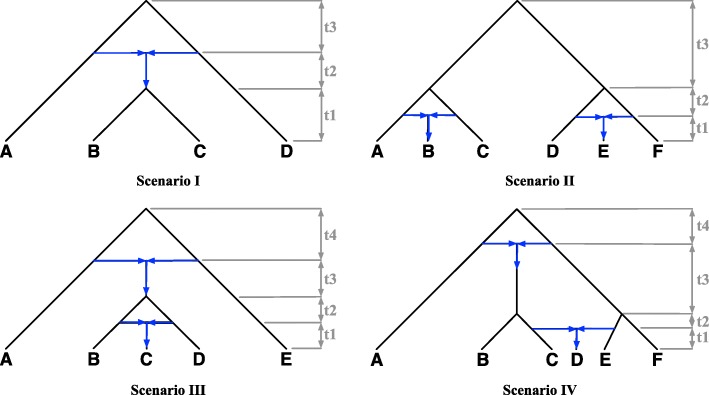
Model phylogenetic networks. Blue arrows indicated directions into and out of the reticulation nodes

**Fig. 3:**
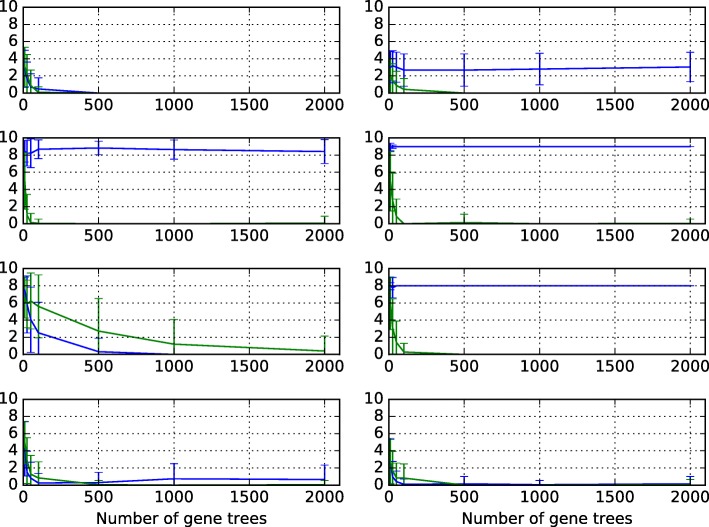
Accuracy of network inference on data simulated under a common mechanism. The symmetric network difference between the inferred and model network, averaged over 100 trials, using the MDC criterion as implemented in [18] (green) and the maximum integrated likelihood under the NCM model (blue). Rows from top to bottom correspond to Scenarios I-IV, respectively, of Fig. 2. Left and right columns correspond to branch length settings 1 and 2, respectively

We first set out to study the performance of maximum integrated likelihood inference under the NCM model, and compare it to that of inference under the parsimony criterion “minimizing deep coalescence" (MDC) of [18]. We follow the same simulation setup, including the model networks, parameters, and numbers of gene trees as that in [18]. More specifically, we considered four phylogenetic network topologies involving distinct combinations of reticulation and speciation events, as shown in Fig. 2. To better understand the effects of deep coalescence in each scenario, we used two settings of the branch length parameters for each network. In branch length setting 1, each of the values *t*1, *t*2, *t*3, and *t*4 are equal to 1 coalescent unit. In branch length setting 2, each value is equal to 2 coalescent units. Setting 1 should involve more deep coalescence events, while setting 2 involves longer branches which are less likely according to the exponential prior on the branch lengths. Each provides unique challenges for the integrated likelihood inference under NCM. All inheritance probabilities were set to 0.5. For each setting and for each number of loci in the set {10,25,50,100,500,1000,2000}, we generated 100 data sets of gene trees using the program ms [21]. It is important to highlight here that the data was generated under a common mechanism; that is, not under the NCM model. We then ran the inference method under MDC of [18] and the maximum integrated likelihood inference under the NCM model on each data set. Since neither the MDC nor the likelihood criteria allow for determining model complexity (in this case, the number of reticulations) in a systematic way, we ran the method here with the maximum number of reticulations set at the true number, which is 1 for the data corresponding to Scenario I, and 2 for the other three scenarios. We then computed the topological distance of [22], as implemented in PhyloNet [23], between each inferred network and the model network on which the data was generated, and averaged the results over all 100 data sets for each setting. The results are shown in Fig. 3. Note that for the calculation of the NCM integrated likelihood, we used a non-truncated exponential prior for the branch lengths of the species phylogeny. This is equivalent to letting the hyperparameter *τ* grow arbitrarily large, and results in a similar likelihood function as that in Eq. (5).

**Fig. 4:**
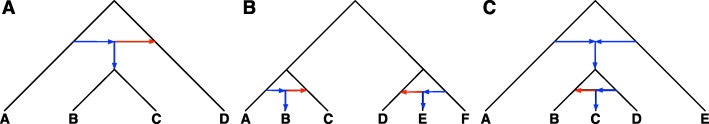
The incorrect networks inferred under the NCM model. While the correct networks were inferred for many data sets, incorrect networks were inferred in other cases, and those incorrect networks are shown in this figure. **a** The network inferred from the data generated on the network of Scenario I and branch length setting 2. **b** The network inferred from the data generated on the network of Scenario II and both branch length settings. **c** The network inferred from the data generated on the network of Scenario III and branch length setting 2. Red arrows indicate the reticulations whose direction was inferred in the reverse order

As the results show, both methods have almost the same behavior and accuracy under branch length setting 1 for the networks of Scenarios I, III, and IV, and under branch length setting 2 for the network of Scenario IV. However, while MDC always converged onto the true network, inference under the NCM model diverged from the true network in the other cases. We then set out to compare the erroneous networks inferred under the NCM model (Fig. 4) to their true counterparts. A quick inspection of the three networks in Fig. 4 points to a very interesting pattern. The only errors in the inferences were the direction of the reticulation edge (as highlighted with red arrows in the figure).When we inspect the three networks in Fig. 4 and their counterparts in Fig. 2, we find that every two corresponding networks in the two figures display the same set of trees. Each of the network corresponding to Scenario I in Fig. 2 and the network in Fig. 4a displays the same two trees: ((A,(B,C)),D) and (A,((B,C),D)). Each of the network corresponding to Scenario II in Fig. 2 and the network in Fig. 4b displays the same four trees (((A,B),C),((D,E),F)), (((A,B),C),(D,(E,F))), ((A,(B,C)),((D,E),F)), and (((A,B),C),(D,(E,F))). Each of the network corresponding to Scenario III in Fig. 2 and the network in Fig. 4c displays the same four trees: ((A,((B,C),D)),E), ((A,(B,(C,D))),E), ((E,((B,C),D)),A), and ((E,(B,(C,D))),A). These pairs of networks are indistinguishable when a single individual per species is sampled, as discussed in [24]. Branch length information on the gene trees and/or sampling multiple individuals per species could resolve this indistinguishability [25], especially as the set of displayed trees does not necessarily characterize a phylogenetic network in the presence of ILS [8].

### Accuracy on data simulated without a common mechanism

**Fig. 5:**
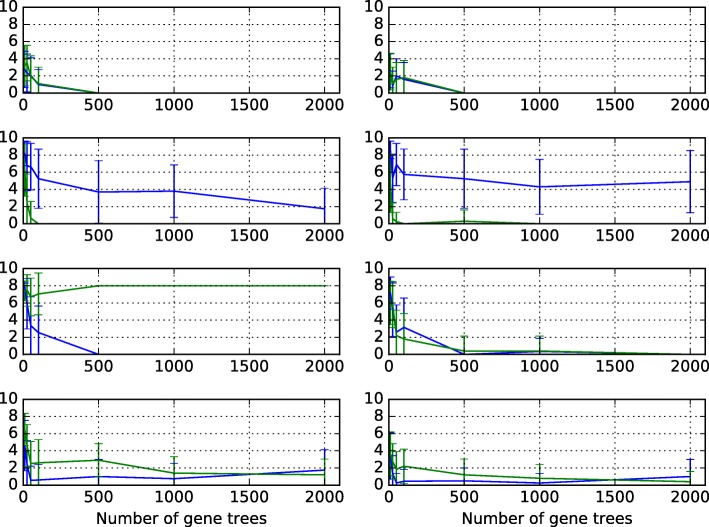
Accuracy of network inference on data simulated under NCM. The symmetric network difference between the inferred and model network, averaged over 20 trials, using the MDC criterion as implemented in [18] (green) and the maximum integrated likelihood under the NCM model (blue). Rows from top to bottom correspond to Scenarios I-IV, respectively, of Fig. 2. Left and right columns correspond to branch length settings 1 and 2, respectively

To study the behavior of inference under an NCM model when the data are evolved without a common mechanism, we again used the network topologies in Fig. 2 to simulate the data. To model a no-common-mechanism evolutionary process, in this experiment, each time a single gene tree was simulated under a network topology, settings for the continuous parameters were sampled from a distribution. As before, to vary the amount of deep coalescence, we used two settings for the branch lengths of each network topology. In branch length setting 1, each of the values *t*1, *t*2, *t*3, and *t*4 are sampled from a uniform distribution from 0 to 2 coalescent units. In branch length setting 2, each value is sampled from a uniform distribution from 0 to 4 coalescent units. In each case, every inheritance probability was sampled from a uniform distribution from 0 to 1. For each setting and for each number of loci in the set {10,25,50,100,500,1000,2000}, we generated 20 data sets of gene trees. We again inferred a network for each collection of data using both MDC and maximum integrated likelihood under NCM, and calculated each average topological difference to the true network. The results are shown in Fig. 5. As the results show, except for Scenario II, inference under the NCM model now improved, and for branch length setting 1 on Scenario III, MDC’s performance became poor.

### Analysis of a mosquito data set

**Fig. 6:**
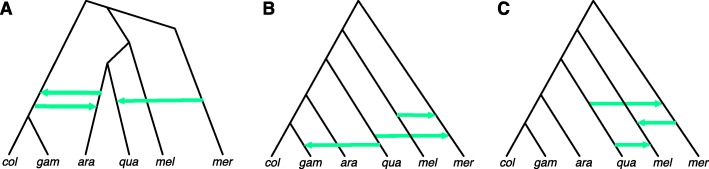
Networks for the mosquito data set. **a** The phylogenetic network reported in [26]. **b** The phylogenetic network analyzed using the maximum likelihood method of [5] and reported in [27]. **c** The phylogenetic network inferred under the NCM model

We reanalyzed the *Anopheles* data of [26]. The data consist of one genome from each of the species *An. gambiae* (gam), *An. coluzzii* (col), *An. arabiensis* (ara), *An. quadriannulatus* (qua), *An. merus* (mer) and *An. melas* (mel). *An. christyi* serves as the outgroup for rooting the gene trees. We used the same set of gene trees from the autosomes that were used in the analyses of [27]. In particular, we used the same set of 669, 849, 564, and 709 loci from the 2L, 2R, 3L, and 3R chromosomes, respectively, and where 100 maximum likelihood bootstrap trees were inferred for each locus and used in the inference. These data are already available in DRYAD, entry doi:10.5061/dryad.tn47c. In [27], the phylogenetic network was inferred from the gene trees using the maximum likelihood method of [5]. The phylogenetic networks from the original study of [26], from the maximum likelihood analysis of [27], and the one we obtained under the NCM model are shown in Fig. 6.

As reported in [27], the likelihood of the network in Fig. 6b was much higher than that of the network reported by Fontaine et al. and shown in Fig. 6a. The log-likelihood under the NCM model of the networks in Fig. 6b and 6c are -17520.49 and -16821.33, respectively. This demonstrates that the difference in the inferred networks is not due to limitations of the search procedure, but due to a better likelihood of the new inferred network over existing ones under the NCM model. It is important to note both networks of Fig. 6b and 6c agree on the same underlying tree structure (the one obtained from the network by removing the green horizontal arrows, and sometimes called the backbone tree). This tree disagrees with that reported by Fontaine et al. [26], and this disagreement was discussed in [27]. Our inferred network also agrees with that of [27] in terms of the *An. quadriannulatus* and *An. merus* hybridization. However, the *An. merus* and *An. melas* hybridization differ in terms of the direction of the reticulation (similar to the trend observed on the simulated data and discussed above), and the *An. quadriannulatus* and *An. melas* hybridization is not reported in [27].

There could be several reasons for the differences between the two networks of Fig. 6b and 6c. The obvious one is that the two networks were inferred under two different models, one that a common mechanism of evolution underlies all loci and the other that assumes each locus has its own model. Second, as discussed in [28], (unpenalized) maximum likelihood cannot determine the correct number of reticulations. It could be that as more complex networks (ones with more than three reticulations) are inferred, the two analyses using the method of [5] and the one under the NCM model might converge onto the same network. These differences notwithstanding, inference under the NCM model recovered a very similar network, which makes it promising to explore the network space first without optimizing or sampling the continuous parameters, and then potentially follow up with a sampling phase to recover parameter values.

### MDC vs. integrated likelihood under nCM

**Fig. 7:**
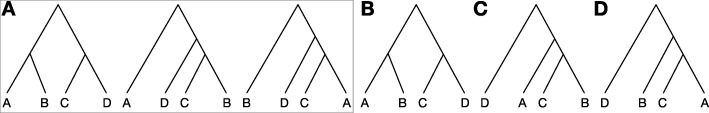
Different optimal species trees under the MDC criterion and NCM model. **a** Three gene trees assumed to have equal frequencies. **b** Optimal species tree under the MDC criterion (has a cost of 4 extra lineages). The other two trees each have a cost of 5 extra lineages. **c** and **d** Optimal species trees under the NCM model

Tuffley and Steel [10] showed that an NCM model is related to Fitch’s parsimony for character evolution in the following way. The maximum likelihood tree (or trees) under their NCM model is also a maximum parsimony tree under certain conditions. In light of this, we investigated whether a similar correspondence might hold for the NCM model for gene trees evolving down a species trees. Using the three gene trees of Fig. 7a, and assuming equal frequencies of all three, we inferred the optimal tree under the MDC criterion as well as the optimal tree under the NCM model. Figure 7b-d shows the results. As the results show, the two criteria in this case result in different optimal species trees. Furthermore, in this case, the optimal tree under the NCM model is not unique.

## Conclusions

In this paper, we introduced a no-common mechanism for phylogenomics, where the species phylogeny topology is the same across all loci, but the gene tree of every locus evolves under its own parameter (branch lengths and inheritance probabilities) settings. We implemented a maximum integrated likelihood function under this NCM model, assessed its accuracy and compared it to inferences under the parsimony MDC criterion on simulated data and the maximum likelihood inference on an empirical data set of mosquito genomes. We found that the inference produces very good results and when there is a disagreement, it is most often due to indistinguishability when using only gene tree topologies and a single individual per species. The main rationale behind developing such a mechanism is to allow for developing methods for efficiently exploring the species phylogeny space while focusing on traversing different topologies without the need for sampling or optimizing branch lengths and other continuous parameters. The running time of calculating the likelihoods (integrated and standard) of the networks in the simulated data above took few than 10 minutes for each network (and the set of gene trees used as the input data). The major gain is in the running time of maximum likelihood inference based on the two methods. The standard maximum likelihood estimation was run for eight hours on each data set, and it took all that time to evaluate candidate networks with their branch lengths. In the case of inference based on the integrated likelihood, and since branch lengths do not factor in the search itself, it took much less time to infer (locally) optimal networks. In fact, if a search technique is designed so as to ensure that the same network topology is not visited more than once during the search (which is not the case in the heuristic employed currently in PhyloNet), search based on the integrated likelihood would be improved much more significantly.

The work gives rise to several questions. First, under which conditions are optimal trees or networks identical under both the MDC criterion and the NCM model? Second, is there a parsimony criterion other than MDC under which the optimal species phylogeny is always identical to the optimal tree under the NCM model? Third, under what conditions, if any, is inference under the NCM model statistically consistent? Fourth, in light of the results above, an important question would be to explore why optimal networks under the NCM model often have reticulations in the opposite direction from those in optimal networks under the likelihood model with a common mechanism across all loci. Answering these and other questions will open up many research avenues in the area of phylogenomics.

Finally, it is important to conduct more studies where different loci have different evolutionary parameters so as to mimic data coming from autosomes and sex chromosomes, as well as loci under selection or where duplication and loss could have played a role. In such cases, assuming a common mechanism underlying all loci is inappropriate and inference under an NCM model could provide more accurate results.

## Data Availability

Not applicable.

## Abbreviations

DAG: Directed, acyclic graph
ILS: Incomplete lineage sorting
MDC: Minimizing deep coalescence
MSC: Multispecies coalescent
NCM: No common mechanism
